# A Nanopore-sequenced high-quality genome of the *Bipolaris oryzae* strain Bo-1

**DOI:** 10.1128/mra.00388-24

**Published:** 2024-10-04

**Authors:** Meng Liu, Wei Yang, Xinyi Gu, Yongxin Xiao, Lei Yang, Zhaowu Zhang, Tong Wei, Tom Hsiang, Yaling Zhang, Guotian Li

**Affiliations:** 1 National Key Laboratory of Agricultural Microbiology, Hubei Hongshan Laboratory, Hubei Key Laboratory of Plant Pathology, The Center of Crop Nanobiotechnology, Huazhong Agricultural University, Wuhan, China; 2 College of Agronomy, Heilongjiang Bayi Agricultural University, Daqing, China; 3 State Key Laboratory of Agricultural Genomics, BGI Research, Shenzhen, China; 4 BGI Research, Wuhan, China; 5 Environmental Sciences, University of Guelph, Guelph, Ontario, Canada; University of Maryland School of Medicine, Baltimore, Maryland

**Keywords:** brown spot disease, *Bipolaris oryzae*, *Oryza sativa*, Nanopore, genome assembly

## Abstract

Brown spot disease of rice caused by *Bipolaris oryzae* results in severe yield losses. A high-quality genome was assembled using Nanopore sequencing data, resulting in a 36-Mb nuclear genome with 19 contigs and a mitogenome. This assembly provides valuable genetic resources for investigations of rice-*B. oryzae* interactions.

## ANNOUNCEMENT

Brown spot disease of rice caused by *Bipolaris oryzae* (1) can lead to over 50% reduction in rice production. Assembled genomes are required for pathogenicity and interaction studies (2). Therefore, we generated a fully assembled genome of *B. oryzae* strain Bo-1.

Isolate Bo-1 was obtained from rice leaves with typical lesions of brown spot disease in a field near Harbin, China. Diseased leaf tissue was sterilized in 2.5% NaClO for 30 s, rinsed using sterile water, and grown on potato dextrose agar (PDA) at 28°C. Hyphae from the edge of a single colony were transferred to fresh PDA and grown for 7 days. The colonized agar was broken into small pieces and cultured in liquid complete medium at 200 rpm, 28°C for 2 days. The hyphae were isolated by filtration and then subjected to DNA extraction and subsequent sequencing. The internal transcribed spacer region was sequenced as previously described (3). The BLAST result suggested that Bo-1 had high homology (99%) with *B. oryzae* (accession no: MT446115.1).

Genomic DNA was extracted from 9-day-old mycelia using a QIAGEN Genomic DNA Extraction Kit. The Nanopore long-read library was constructed using sheared DNA > 23 kb in length with a ligation sequencing kit (SQK-LSK110) and sequenced on the PromethION flow cell (R10.4.1). Using Guppy version 6.2.1 (4) rapid base calling to trim adapters and retain reads of quality score ≥ 7, we obtained 391,025 long reads (*N*_50_ = 27,805 bp). The short-read library was constructed using the MGIEasy DNA Library Prep Kit and processed with an MGI DNBSEQ-T7, and after filtering with fastp version 0.23.4 (5), this generated 22,141,120 150-bp paired-end reads. For RNA-seq, total RNA was extracted from 9-day-old mycelia with the TRIzol reagent. The RNA library was constructed using the MGIEasy RNA Library Prep Kit and sequenced on the MGI DNBSEQ-T7. We obtained 25,389,448 150-bp paired-end RNA-seq raw reads and 25,383,914 clean reads after filtering reads with *q* value < 20 with fastp.

We assembled the genome using NextDenovo version 2.5.2 (6) with long reads. The draft genome was polished for three rounds using NextPolish version 1.4.1 (7) with short reads and long reads. The final 36-Mb genome was assembled into 19 contigs (*N*_50_ = 2.40 Mb). Repetitive sequences were identified with RepeatMasker version 4.1.2 (8) and RepeatModeler version 2.0.5 (9). Finally, BUSCO version 5.5.0 (10) analysis showed that 99% of BUSCO genes were identified by using the fungi_odb10 database.

Protein-coding genes were predicted by Funannotate version 1.8.15 from RNA-seq reads and functionally annotated with Eggnog-mapper (11). Secondary metabolite biosynthetic gene clusters were identified using antiSMASH (12). SignalP version 5.0b (13) and TMHMM version 2.0c (14) were used to identify secreted proteins. EffectorP version 3.0 (15) was used to identify predicted effectors. We identified carbohydrate enzymes using dbCAN2 (16). Genomic information is presented in Table 1.

**TABLE 1:** Summary of the *Bipolaris oryzae* genomes between Bo-1 and ATCC 44560^*a*^

Characteristics	Strain	
	Bo-1	ATCC 44560
Sequencing technology	ONT, MGI	Illumina
Sequencing depth	253×	191×
Genome size (bp)	35,993,084	32,695,608
Number of contigs	19	671
Contig *N*_50_ (bp)	2,397,707	134,117
Maximal contig length (bp)	3,604,784	638,489
GC content (%)	49.87	50.50
Repeat sequence (bp)	5,323,340	NA
BUSCO completeness (%)	99	99
Predicted genes	10,633	12,002
Secreted proteins	843	NA
Potential effectors	326	NA
Carbohydrate enzymes	565	NA
Mitogenome size (bp)	130,988	124,887

^*a*^ NA, not available.

**Fig 1:**
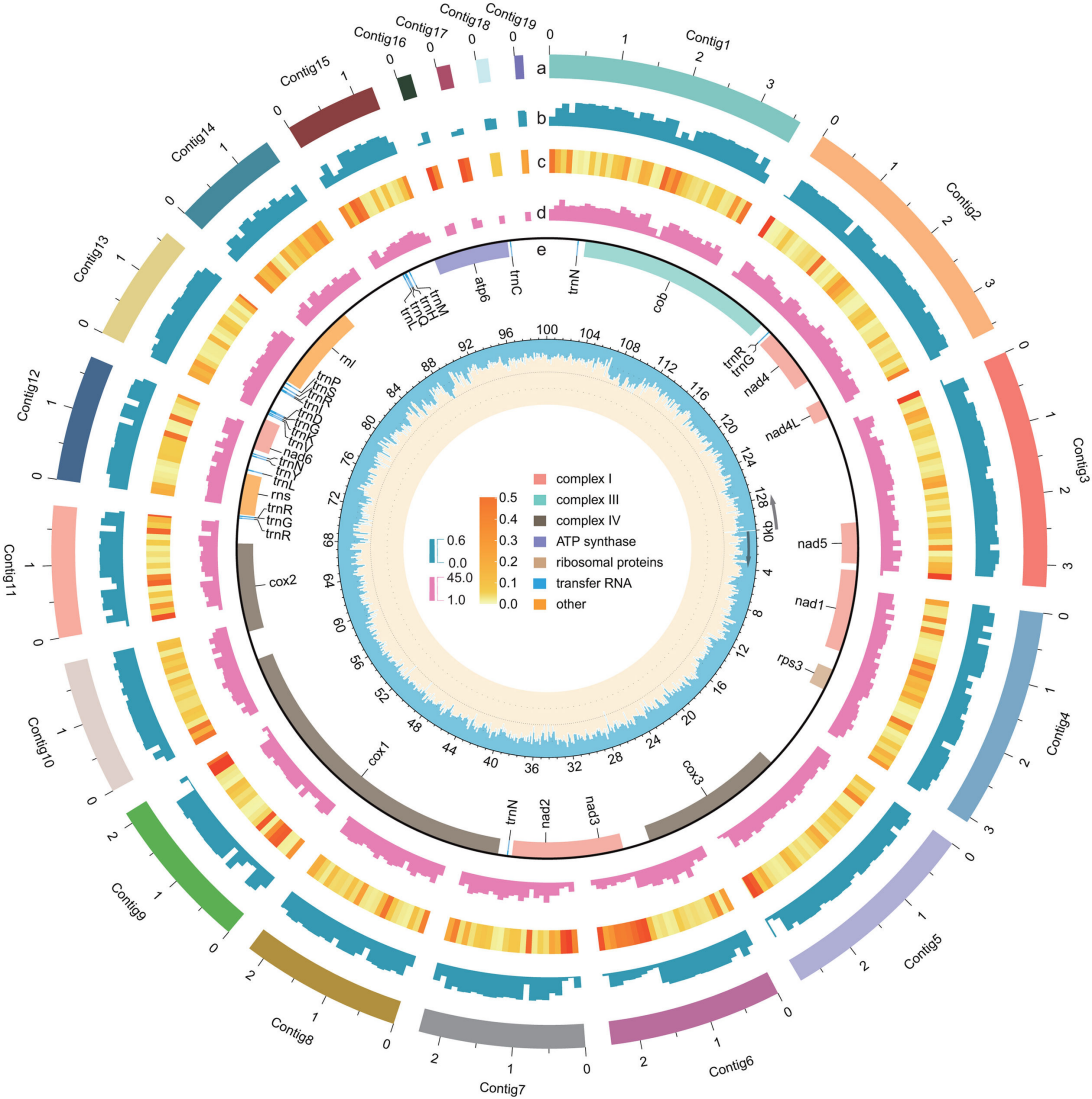
Nuclear and mitochondrial genomes of *B. oryzae* strain Bo-1. The 19 contigs on a megabase-pair scale. b, GC content. c, Repeat density. d, Gene density. The Circos plot shows the nuclear genome at non-overlapping 100-kb intervals in a, b, c, and d. e, Mitogenome. The outer circle indicates the distribution of annotated genes. The inner circle indicates GC content (blue), which was calculated in non-overlapping 100-bp intervals.

Based on short reads, the 130,988-bp circular mitogenome with a 30.17% GC content was assembled with GetOrganelle version 1.7.7.0 (17). Moreover, 13 protein-coding genes, 23 tRNAs, and 2 rRNAs were annotated with GeSeq (18). TBtools version 2.026 (19) and Chloroplot (20) were used to visualize the nuclear and mitochondrial genomic features, respectively (Fig. 1).

## Data Availability

Software parameter commands are uploaded to the Figshare database (https://doi.org/10.6084/m9.figshare.24884610.v2), while default parameters are used for unspecified software. The Bo-1 genome file is available in the NCBI database under the project accession number PRJNA1054994. The sequencing raw reads of Nanopore long reads, short reads, and RNA-seq reads have been deposited under the accession numbers SRR27467475, SRR27467476, and SRR27322759 in the SRA database. The genome sequences have been deposited at GenBank under the accession number GCA_037177745. The nuclear and mitochondrial genome assembled and annotated file, predicted carbohydrate enzymes, and effectors have been deposited to the Figshare database (https://doi.org/10.6084/m9.figshare.24884610.v2).
